# Case Report: Lewy body dementia with unusual psychotic symptoms, atypically late parkinsonism, and patient sensitivity to first generation antipsychotics

**DOI:** 10.3389/fpsyt.2025.1551581

**Published:** 2025-04-09

**Authors:** Lolita Ercika, Maris Taube

**Affiliations:** ^1^ Faculty of Residency, Riga Stradiņš University, Riga, Latvia; ^2^ Department of Depression and Crisis, National Center of Mental Health, Riga, Latvia; ^3^ Department of Psychosomatic Medicine and Psychotherapy, Riga Stradiņš University, Riga, Latvia

**Keywords:** dementia, Lewi bodies, psychosis, antipsychotics, sensitivity

## Abstract

Lewy body dementia is associated with abnormal eosinophilic A-synuclein neural inclusions (Lewy bodies) in the brain. It is a neurodegenerative illness—and the second most common type of dementia after Alzheimer’s disease—that causes memory loss and severe problems in carrying out daily activities. In this report, we describe a case of Lewy body dementia that began with early psychotic symptoms with atypical features (transition from hallucinosis (hallucinatory insight) to true visual hallucinations) —without Parkinsonism. The patient exhibited sensitivity to first generation antipsychotic medication, which led to a worsening of her symptoms. Physicians should consider all possible diagnoses when confronted with atypical, early symptoms of visual hallucinosis or true hallucinations and dementia without Parkinsonism. Choosing antipsychotic medicines should be made with care given these patients’ possible sensitivity to antipsychotics. The selection of antipsychotics should be consider among first, second and third generation options.

## 1 Introduction

Dementia with Lewy bodies is the second most common form of neurodegenerative illness with an age of onset ranging between 50 and 80 years (1). This neurodegenerative illness is similar to Parkinson’s disease. Consensus diagnostic criteria for dementia with Lewy bodies are divided in to the Central features (progressive dementia severe enough to interfere with normal social function, deficits of attention, executive function, and visuospatial ability), Core features (fluctuating cognition, recurrent visual hallucinations, spontaneous parkinsonism), Suggestive features (rapid eye movement sleep behavior disorder, severe sensitivity to antipsychotics, low dopamine transporter uptake in the basal ganglia), Supportive features (repeated falls and syncope, transient unexplained loss of consciousness, severe autonomic dysfunction, non-visual hallucinations, systematized delusions, depression, relative preservation of medial temporal lobe structures, generalized low uptake on single photon emission computer tomography (CT) perfusion or positron emission tomography (PET) metabolism with reduced occipital activity, abnormal metaiodobenzylguanidine myocardial scintigraphy, prominent slow wave activity on electroencephalogram with temporal lobe transient shape waves (2, 3).

Dementia with Lewy bodies is often misdiagnosed as Alzheimer’s disease or Parkinson’s disease dementia (4). Visual hallucinations, parkinsonism, and fluctuating cognition are specific signs of mild to moderate dementia with Lewy bodies. These symptoms are typical of Alzheimer’s disease in later stages of dementia with Lewy bodies as well and complicate diagnostics (5).

Lewy body dementia has a poor prognosis, rapid cognitive impairment, and a significant negative impact on patient quality of life (6).

This particular case is interesting with early psychotic symptoms with atypical features (transition from hallucinosis (hallucinatory insight) to true visual hallucinations) with late and atypically long-time development of Parkinsonism.

From the perspective of the clinical diagnostic approach, it is crucial to consider misperceptions such as pareidolias and visual nonthreatening hallucinations, particularly in the early stages of the disease, even if parkinsonism is not present.

## 2 Case description

In 2024 a 91-year-old woman was hospitalized for a second time in his life after she was found at home lying on the floor, confused and speech impaired. Symptoms development during the years are described in the **Table 1**.

**Table 1:** Symptoms development timeline.

Year	Clinical description of the patient’s condition
2009	Experiencing visual hallucinations, which she realized were not real, was seeing her mother, who visited her in the evenings despite the patient’s mother having died
2010/2011	Unable to distinguish hallucinations from reality, and her cognitive condition, e.g. memory function worsened
2021	Became disoriented, confused the names of relatives, refused to eat, slept for long periods, and remained out of contact with others, was transferred to the nursing home
2024	Understood questions asked of her, but her answers are often approximate, indirectly descriptive, and redundant. She did not remember her name but remembered her daughter’s last name. She understood that she was in a hospital but was disoriented in time and did not fully understand her situation

Medical history obtained from the patient’s son revealed that approximately 15 years ago, the patient began experiencing visual hallucinations, which she realized were not real. At the time, the patient told her son she was seeing her mother, who visited her in the evenings despite the patient’s mother having died. As the patient’s mental condition worsened, she was unable to distinguish hallucinations from reality, and her cognitive condition, e.g. memory function worsened. The patient’s first hospitalization occurred in 2021, when she became disoriented, confused the names of relatives, refused to eat, slept for long periods, and remained out of contact with others. During this period, the patient was transferred to a nursing home, where she gradually resumed eating and walking, and her consciousness cleared.

In 2024, the patient returned to the hospital with complaints similar to the ones she had in 2021. This time, her interactions with other patients in the hospital was satisfactory. She usually understood questions asked of her, but her answers were often approximate, indirectly descriptive, and redundant. She did not remember her name but remembered her daughter’s last name. She understood that she was in a hospital but was disoriented in time and did not fully understand her situation. On some days, she thought she was traveling by train, and on other days she believed it was Christmas. She willingly engaged in conversations and was polite and sincere. Her speech was rapid, sometimes poorly modulated, and difficult to understand. She often forgot words and spoke using descriptive phrases. When asked about berries she said she had picked that day, she might reply that she really picked grapes, then change her mind and say someone else picked them and brought them to her, and then change her mind again and declare that she ‘picked them in her sleep, in a dream’. In general, the patient was unconcerned about her perceptions and memory disorder. Her mood was slightly elevated. Her thought patterns were medium-paced, although sometimes she would exhibit obsessive, repetitive thinking, or she would lose her train of thought. At times she could also be illogical and contradictory.

## 3 Diagnostic assessment

A diagnosis of Lewy body dementia is made in this particular case based on consensus criteria that includes progressive dementia severe enough to interfere with social and occupational functioning, with attention and executive function damage (3). Recurrent visual hallucinations, cognitive fluctuations, and electroencephalography with temporal lobe transparent sharp waves suggested a diagnosis of Lewy body dementia in this patient. However, the late onset of Parkinsonism is atypical for this diagnosis (2).

From a neurological perspective, the patient demonstrated a positive bilateral palmomental reflex, which may indicate frontal lobe dysfunction. A positive Babinski’s reflex on the right side was also observed. On the left side of the body, there was slight stiffness in the arm and leg, and the cogwheel was observed in the left hand. No resting tremors were observed.

The patient was given 5 mg olanzapine tablets once daily and five drops of 0.2% haloperidol oral solution twice daily, mostly to address hallucinations.

However, she exhibited a pronounced sensitivity to antipsychotics and became rigid, drowsy, and withdrawn.

Due to these side effects, the patient underwent a computed tomography scan of the brain. There was no evidence of acute ischemia, although vascular encephalopathy was noted. To reduce the side effects, the haloperidol was removed from her treatment regime.

A lumbar puncture was performed on the patient. No cytosis was detected, but Tau protein levels were elevated, indicating neurodegeneration. The patient also underwent a computed tomography scan of the brain. There was no evidence of acute ischemia, although vascular encephalopathy was noted.

The results of examinations performed in an inpatient neurological setting are presented in **Table 2**.

**Table 2:** The results of examinations performed in an inpatient neurological setting.

Date	Examination	Results
11/01/2024	Magnetic Resonance Imaging (MRI) of the head	In the parenchyma of the brain, acute ischemic or hemorrhagic pattern changes are not seen. Diffuse moderate enlargement of the cerebral sulci and ventricles. Vascular leukoencephalopathy. Chronic atherosclerosis of the main intracranial arteries.
29/12/2023	Electroencephalography (EEG)	The EEG is dominated by general changes: a slowed-down theta registers, an alpha base rhythm with a frequency of 7-8-9 Hz, without regional zoning. In the temporal areas of both hemispheres, less pariental, with lanternization in the right hemisphere, a more pronounced, regional deceleration registers. Epileptiform activity is not recorded in the EEG, findings are consistent with encephalopathy (dementia)
28/12/2023	Liquor (only abnormal results)	Tau protein 789 pq/mL (reference interval 0-290) pTau181 >112 pq/mL (phosphorylated tau protein) (reference interval 0-60) AB42 (beta amyloid 42) 771 pq/mL (reference interval >630) Protein qualitative 0.520 g/L (reference interval > 0.2-0.4) Glycoses ‘level 4 mmol/l (reference interval 2,2-3,9)
22/12/2023	Computer tomography (CT)	Brain and skull bones without CT visualizable acute pathology. Vascular encephalopathy.

In the hospital, the patient’s condition remained stable. Given that she has pronounced cognitive deficits and requires help caring for herself, she was transferred to a long-term care facility.

## 4 Discussion

Lewy body dementia is associated with abnormal eosinophilic A-synuclein neural inclusions (Lewy bodies) in the brain (7). An official diagnosis is made through an autopsy (6). New methods, like skin biopsy are introduced and will be performed in the future (8). We performed a lumbal puncture and found elevated TAU proteins suggestive of the disease. Lewy body dementia patients present with a fluctuating cognitive state, with neuropsychiatric, sleep, motor, and autonomic symptoms (9). In this case, the patient experienced hallucinations 15 years before present hospitalization in 2024 and severe dementia. Furthermore, the course of her disease fluctuated.

Visual hallucinations in the case of dementia with Lewy bodies are usually well formed and featured people, children, or animals (4). Visual hallucinations in early dementia are highly specific for a diagnosis of dementia with Lewy bodies (10). The hallucinations in dementia with Lewy bodies usually are nonthreatening misperceptions of ambiguous stimuli, termed pareidolias (11). In contrast in Alzheimer’s disease, hallucinations generally have a threatening quality (12). Diagnosis of dementia with Lewy bodies is less likely if parkinsonism does not develop until severe dementia (2).

In this patient, the initial dose of haloperidol may have been too high. Given the patient’s age, typical antipsychotics should have been introduced gradually via slow, daily up titration. Moreover, patients with Lewy body dementia may be sensitive to antipsychotic drugs, which can cause serious side effects, such as worsening extrapyramidal symptoms, aggression, and agitation (13, 14). For this patient 5 mg Olanzapine tablets taken orally once a day was sufficient. Risk of extrapyramidal symptoms are higher with typical antipsychotics and higher potency atypical antipsychotics, such as olanzapine and risperidone (15) Using typical antipsychotics, such as haloperidol, was an dangerous decision in this case. If nonpharmacological treatments fail to address aggressive behavior or related distress, haloperiodol will be used; however, safer alternatives, such as aripiprazole, will be taken into consideration (16). Cholinesterase inhibitor therapy is safe and effective treatment for psychotic symptoms in dementia with Lewy bodies (11, 17).

### 4.1 Limitations

The development of hallucinations in our patient may be associated with the development of other prevailing vascular processes. Moreover, the diagnosis of Lewi body dementia was clinical. The presence and severity of the symptoms was evaluated by a clinician based on their own experience and knowledge, and the diagnosis was not confirmed histologically. Various factors (vascular and metabolic) can interact during the development of dementia; therefore, patient reactions to medicines may be related to processes other than those related to Lewy body dementia.

## 5 Conclusion

In this patient, her disease presented with early psychotic symptoms— atypical visual hallucinations with specific development from hallucinosis till true visual hallucinations without Parkinsonism. Physicians should consider all possible diagnoses when confronted with atypical, early symptoms of dementia without Parkinsonism and with slow decrease of cognitive function in atypically long period of time. Choosing antipsychotic medicines should be made with care given these patients’ possible sensitivity to antipsychotics.

## 6 Patient perspective

Currently, the patient is receiving complete care in a social care institution. Her cognitive functions, based on the opinion of her relatives, have not improved, and she is experiencing movement difficulties.

## Data Availability

The original contributions presented in the study are included in the article/supplementary material. Further inquiries can be directed to the corresponding author.
